# miRNAs as biomarkers of therapeutic response to HER2-targeted treatment in breast cancer: A systematic review

**DOI:** 10.1016/j.bbrep.2023.101588

**Published:** 2023-11-28

**Authors:** Thanh Hoa Vo, Esam EL-Sherbieny Abdelaal, Emmet Jordan, Orla O'Donovan, Edel A. McNeela, Jai Prakash Mehta, Sweta Rani

**Affiliations:** a Department of Science, School of Science and Computing, South East Technological University, Cork Road, Waterford, X91 K0EK, Ireland; b Pharmaceutical and Molecular Biotechnology Research Center, South East Technological University, Cork Road, X91 K0EK, Waterford, Ireland; c Department of Oncology, UPMC Whitfield Hospital, Cork Road, X91 DH9W, Waterford, Ireland; d Department of Oncology, University Hospital Waterford, Dunmore Road, X91 ER8E, Waterford, Ireland; e Department of Applied Science, South East Technological University, Kilkenny Road, R93 V960, Carlow, Ireland

**Keywords:** HER2-Positive breast cancer, miRNAs, Treatment response, HER2 target therapy, Drug resistance, Drug sensitivity

## Abstract

Breast cancer is the most common type of lethal cancer in women globally. Women have a 1 in 8 chance of developing breast cancer in their lifetime. Among the four primary molecular subtypes (luminal A, luminal B, HER2+, and triple-negative), HER2+ accounts for 20–25 % of all breast cancer and is rather aggressive. Although the treatment outcome of HER2+ breast cancer patients has been significantly improved with anti-HER2 agents, primary and acquired drug resistance present substantial clinical issues, limiting the benefits of HER2-targeted treatment. MicroRNAs (miRNAs) play a central role in regulating acquired drug resistance. miRNA are single-stranded, non-coding RNAs of around 20–25 nucleotides, known for essential roles in regulating gene expression at the post-transcriptional level. Increasing evidence has demonstrated that miRNA-mediated alteration of gene expression is associated with tumorigenesis, metastasis, and tumor response to treatment. Comprehensive knowledge of miRNAs as potential markers of drug response can help provide valuable guidance for treatment prognosis and personalized medicine for breast cancer patients.

## Highlights

• A systematic review on the role and challenges of miRNAs in predicting responses to HER-targeted therapy in breast cancer.
• A summary of the mechanisms involved in miRNA-mediated HER2 therapy resistance.
• Discussing future use of miRNA biomarkers in HER2 treatment selection for poor responders to current HER-targeted therapies.

## Graphical abstract

**Figure:**
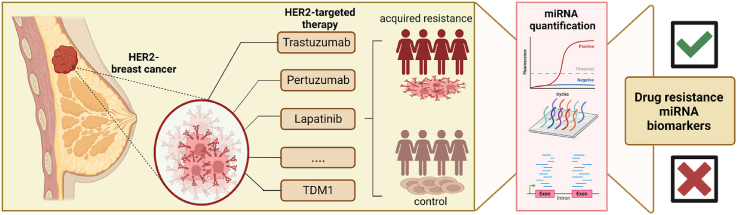

## List of abbreviations

ADC: antibody-drug conjugates
ADCC: antibody-dependent cellular cytotoxicity
ATG5: Autophagy related 5
CCNJ: cyclin J
CDK: cyclin-dependent kinase
CNS: central nervous system
CSCs: cancer stem cells
EGFR: epidermal growth factor receptor
EMT: epithelial-to-mesenchymal transition
ERKs: extracellular signal-regulated kinases
FOXO3a: forkhead box O3a
FUBP1: far upstream element-binding protein 1
H1F-1α: hypoxia-inducible factor 1-alpha
IGF1R: insulin-like growth factor-I receptor
IkB-α: nuclear factor of kappa light polypeptide gene enhancer in B-cells inhibitor, alpha
IRS1: insulin receptor substrate 1
MAPK: mitogen-activated protein kinase
miRNA: microRNA
mTOR: mammalian target of rapamycin
mTORC2: mTOR complex 2
NF217: Krüppel-like zinc finger protein 217
pAKT: phosphorylated AKT
PDCD4: programmed cell death 4
PI3K: phosphatidylinositol 3-kinase
PLCXD1: phosphatidylinositol-specific phospholipase-C-X-domain-containing-1
PLCγ: phospholipase C gamma
PTEN: phosphatase and tensin homolog
STAT3: signal transducer and activator of transcription 3
VEGF: vascular endothelial growth factor
ZEB1: zinc finger E-box binding homeobox 1

## 1 Background

Breast cancer is the most common type of lethal cancer in women globally. Women have a 1 in 8 chance of developing breast cancer in their lifetime [1]. Among the four primary molecular subtypes (luminal A, luminal B, HER2-positive, and triple negative), HER2-positive (HER2+) breast cancer is considered rather aggressive and accounts for 20–25 % of all breast cancer [2,3]. Human epidermal growth factor receptor 2 (HER2) is a tyrosine kinase receptor encoded by the ERBB2 gene in humans. HER2 belongs to the human epidermal growth factor receptor family and is involved in signal transduction pathways modulating cell growth and differentiation. HER2 can dimerize with other HER family receptors [4]; creating the long-lived potent HER2-involving heterodimerization. When HER2 is overexpressed, cell growth and differentiation are facilitated through the activation of phosphatidylinositol 3-kinase (PI3K)/protein kinase B (AKT) and the Ras/Raf/mitogen-activated protein kinase (MAPK)/extracellular signal-regulated kinases (ERKs) pathways [5]. The number of HER2 proteins displayed by HER2+ cancer cells is roughly one hundred times higher than that of normal cells [6]. The amplification of HER2 on the cell surface of malignant breast tumors represents a strong predictor of poor prognosis [7,8].

The era of targeted therapeutic interventions for HER2+ breast cancer began with the Food and Drug Administration's (FDA) approval of the monoclonal antibody trastuzumab in 1998 [9]. Over the years, the list of approved HER2-targeted therapeutic agents has markedly expanded with the inclusion of pertuzumab [10]; trastuzumab emtansine [11]; tyrosine kinase inhibitors like lapatinib [12]; neratinib [13]; tucatinib [14]; and most recently, trastuzumab-deruxtecan [15]. Although the treatment outcome of HER2+ breast cancer patients has been significantly improved with anti-HER2 agents, it is approximated that one in four early-stage breast cancer patients treated with trastuzumab will relapse within a decade [16] and only 18 months of median progression-free survival was recorded when patients have a triple combination of pertuzumab, trastuzumab and docetaxel [17]. Primary and acquired drug resistance present substantial clinical issues, limiting the benefits of treatment. Drug resistance mechanisms in HER2+ breast cancer have been discussed in a number of reviews [4,[18], [19], [20]]. In this context, the abnormal microRNA (miRNAs) expression leading to anti-HER2 therapeutic resistance has also been investigated [21].

miRNAs are single-stranded, non-coding RNAs of around 20–25 nucleotides, known to regulate gene expression at the post-transcriptional level [22]. Lin-4 was the first discovered miRNA in 1993 [23]. Later, many miRNAs in humans and plants were discovered, and nowadays, over 2000 mature miRNA sequences have been illustrated as having the ability to regulate the expression of about one-third of genes in the human genome [24,25].

Increasing evidence has demonstrated that the alteration of gene expression in tumorigenesis, metastasis, and treatment response of tumors is related to miRNA-mediated regulation [26]. In breast cancer, associated miRNAs can be further subdivided into oncogenic and tumor suppressor miRNAs. The dysfunction of miRNAs has an essential role in pathological processes implicated in the progression of tumors and therapeutic resistance of breast cancer [27]. Comprehensive knowledge of miRNAs as potential markers of drug response can help provide valuable guidance for treatment prognosis and personalized medicine.

In this review, we focus on mechanisms of current HER2-targeted therapeutic drugs, functional mechanisms of miRNAs involved in therapeutic responses and drug resistance to anti-HER2 therapies.

## 2 Selection method

We referred to the Preferred Reporting Items for Systematic Reviews and Meta Analyses (PRISMA) guideline for study selection [28]. We searched four electronic databases up to May 2023: Cochrane Library, PubMed, EMBASE, and Scopus. The search strategy combined the Medical Subject Headings terms and free terms “HER2-positive breast cancer” or “HER2 positive breast cancer”, in combination with “drug resistance”, “drug sensitivity” or “target therapy resistance”, “target therapy sensitivity” or “targeted therapy resistance”, “targeted therapy sensitivity” or “A resistance”, “A sensitivity” (which A are names of HER2 targeted therapy drugs for breast cancer) and “miRNA” or “microRNA”. The eligibility criteria consisted of miRNA biomarkers for targeted drug response in HER2+ breast cancer.

The articles were screened and selected independently by two reviewers, and disagreements were resolved by the third reviewer. All the retrieved publications were entered into reference-manager software (EndNote 20, Excel 2016).

**Fig. 1:**
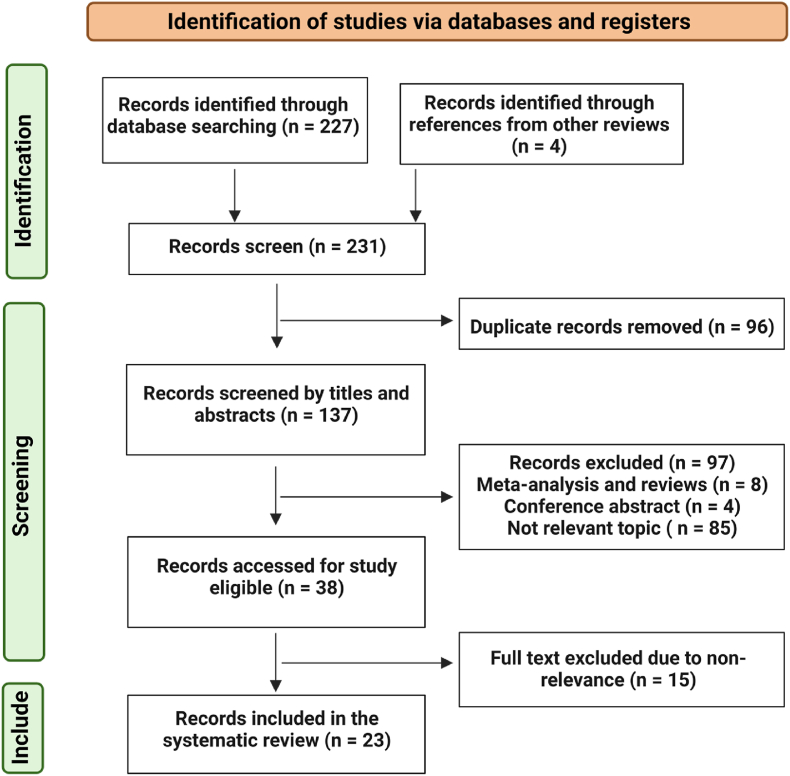
**Selection method**. PRISMA diagram of our literature search strategy.

We identified 231 records from Cochrane Library, PubMed, EMBASE, Scopus database, and reference lists of review articles. After removing 96 duplicates, 97 records were excluded according to the screening of titles and abstracts. Of 38 remaining research studies, 15 studies were removed after evaluating the selection criteria [1]: related to HER2+ breast cancer [2], related to HER2-targeted therapy resistance or sensitivity in breast cancer [3], microRNA. As a result, we had 23 different research studies. Fig. 1 shows the flow diagram of the systematic search.

## 3 HER target therapies

Different types of drugs targeting the HER proteins have been developed and can be characterized into three main types: monoclonal antibodies, antibody-drug conjugates, and tyrosine kinase inhibitors.

### 3.1 Monoclonal antibody

**Fig. 2:**
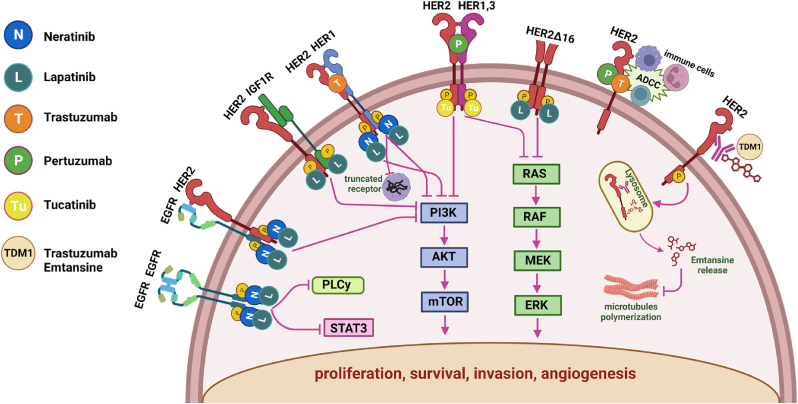
**HER-therapy mechanism of action**. Dimerization of HER2 receptors with other HER receptors activates downstream signaling pathways that modulate proliferation, survival, invasion, and angiogenesis. Trastuzumab binds to epitope IV and prevents HER2 activation, resulting in internalisation and degradation of the protein. Pertuzumab binds to epitope II, preventing homo- and heterodimerization. Trastuzumab and Pertuzumab recruit immune cells to tumor sites that overexpress HER2. Lapatinib reversibly binds to EGFR and HER2, whereas neratinib irreversibly binds to EGFR, HER2, and ERBB4. TKIs bind to the ATP-binding domain of protein kinases to prevent their phosphorylation and block subsequent activation of downstream pathways.

Trastuzumab was the first FDA-approved monoclonal antibody targeting HER2 protein (Fig. 2). It can be used in both early-stage and advanced breast cancer. Several mechanisms of trastuzumab in the treatment of HER2+ breast cancer have been discovered. Proteolytic cleaving of overexpressed HER2 can lead to extracellular domain release [29]. This process frees a more oncogenic truncated membrane receptor compared to the full-length and facilitates the growth and survival of cancer cells [30]. Trastuzumab can act as a HER2 cleavage inhibitor by blocking the metalloproteinase activity to inhibit HER2 extracellular domain shedding [31]. Another proposed mechanism of trastuzumab is inhibiting PI3K/AKT pathways to promote apoptosis and cell proliferation arrest [32,33]. Furthermore, AKT reduces p27kip1, a cyclin-dependent kinase (CDK) inhibitor, by promoting proteolysis and lowering transcription [34]. Some studies have also demonstrated the mechanism of trastuzumab in cell cycle arrest at G0/G1 phase via the activation of p27kip1 [[35], [36], [37]]. HER2 overexpression is also associated with angiogenesis and vascular endothelial growth factor (VEGF) expression. Trastuzumab inhibits VEGF expression by inhibiting PI3K/AKT pathways, thereby suppressing tumor growth [38]. In addition, as an antibody, trastuzumab works through a process known as antibody-dependent cellular cytotoxicity (ADCC), which involves recruiting immune cells to tumor sites [39] (Fig. 2). Using samples from patients with locally advanced breast cancer, Arnould et al. (2006) performed immunohistochemical analysis of immune cell infiltration. Increased immune cells, including T cells, B cells and natural killer cells were observed in tumor infiltrate after trastuzumab and docetaxel treatment [40].

Pertuzumab can be prescribed with trastuzumab and chemotherapy to treat early-stage or advanced HER2+ breast cancer. The extracellular domain of HER2 consists of I, II, III, and IV functional domains and domain II is required for dimerization [41]. While trastuzumab binds to domain IV of HER2, pertuzumab binds to domain II and inhibits ligand-induced HER2/HER3 dimerization [42]; and promotes ADCC [43,44]. It has also been reported that the combination of trastuzumab and pertuzumab could increase apoptosis and the arrest of proliferation in HER2-overexpressed breast cancer cell lines [45].

### 3.2 Antibody-drug conjugates

Antibody-drug conjugates (ADC) are monoclonal antibodies attached to chemotherapeutic drugs. In this case, the anti-HER2 antibody helps direct the chemotherapeutic agents to the cancer cells. Trastuzumab emtansine links HER2 antibody trastuzumab to the chemo drug emtansine. This drug is often used in early-stage breast cancer after surgery or advanced breast cancer after treatment with trastuzumab and chemotherapy. Most recently, in August 2022, FDA approved ADC trastuzumab deruxtecan, which is a trastuzumab linked deruxtecan chemo agent, for the treatment of patients with unresectable or metastatic HER2-low breast cancer. HER2-low breast cancer reflects a group of cancers with substantial biological heterogeneity and with a HER2 immunohistochemical (IHC) score of 1+ or 2+ and a negative in situ hybridization (ISH) result [46].

### 3.3 Tyrosine kinase inhibitors

Tyrosine kinases are significant in modulating the signaling cascade in many biological processes such as growth, metabolism, differentiation, and apoptosis. Another FDA approved (2007) HER-targeting drug for metastatic cancer is lapatinib, a kinase inhibitor of both the epidermal growth factor receptor (EGFR) and HER2 [47] (Fig. 2). It is usually used in combination with trastuzumab. Lapatinib inhibits MAPK, PI3K-AKT, and phospholipase C gamma (PLCγ), which are the main downstream signaling pathways of EGFR and HER2 [48]. Also, lapatinib can inhibit p95HER2, an amino terminally truncated receptor conferring trastuzumab resistance, in vitro and in vivo [49]. Lapatinib blocked the crosstalk between HER2 and IGF-1R in trastuzumab-resistant, SKBR3 cells, preventing HER2 receptor phosphorylation [50,51]. HER2 overexpression induces nuclear factor kappa light chain enhancer of activated B cells (NF-κB) activation inducing radiotherapy resistance [52]. Lapatinib can suppress NF-κB activation by blocking the PI3K/AKT cascade, thereby decreasing phosphorylation of nuclear factor of kappa light polypeptide gene enhancer in B-cells inhibitor, alpha (IkB-α), an inhibitor of NF-κB [53]. Lapatinib treatment inhibits activation of the MEK/ERK signaling pathway, radiosensitising EGFR+ and HER2+ breast cancer cells [54]. Lapatinib achieved antimotility action in SKBR3 cells by upregulating miR-575 and miR-1225-5, and downregulating their common target oncogenic protein PLCXD1 (phosphatidylinositolspecific phospholipase-C-X-domain-containing-1) [55].

Neratinib is a kinase inhibitor of EGFR, HER2, and HER4 which inhibits oncogenic signaling through the MAPK and AKT pathways by blocking the receptors from being phosphorylated at tyrosine residues [56] (Fig. 2). Some clinical trials showed that compared to lapatinib, neratinib demonstrated promising central nervous system (CNS) penetration and treatment effectiveness in those developing brain metastases, although at the cost of a higher incidence of diarrhea as a side effect [57,58]. Neratinib is prescribed to treat early-stage breast cancer after patients have been treated with trastuzumab or to treat metastatic breast cancer when given together with the chemotherapeutic drug capecitabine.

Another FDA-approved kinase inhibitor used to treat unresectable/metastatic (including CNS metastasis) HER2+ breast cancer is tucatinib, usually given in combination with trastuzumab and capecitabine. Tucatinib is known to inhibit the phosphorylation of HER2 and HER3, inhibiting MAPK/AKT signaling and causing cell proliferation changes [14].

## 4 Mechanisms involved in miRNA-mediated HER2 therapy resistance

Several mechanistic studies have described miRNAs as regulators of pathways implicated in the resistance of anti-HER2 therapies. While lack of target dependency or enhancement of compensatory pathways can cause primary resistance, acquired resistance is usually the result of loss of target expression due to continuous treatment or activation of mutations or mechanisms that can enhance cell proliferation [59]. Resistance to HER-targeted therapies can occur due to changes in receptor or downstream signaling components [60].

### 4.1 PI3K/AKT/mTOR signaling pathway

The PI3K/AKT/mammalian target of rapamycin (mTOR) intracellular signaling pathway plays a significant role in regulating the cell cycle. The direct relation of this pathway in cancer is modulated via various activities like cell growth, proliferation, apoptosis, or angiogenesis [61]. Under PI3K activation, AKT is recruited to the plasma membrane and phosphorylated by mTOR complex 2 (mTORC2). Phosphorylation of target cell membrane proteins by activated AKT stimulates cell survival, growth, and proliferation [62]. PI3K/AKT mutations, such as PIK3CA mutations or amplifications, and PTEN (phosphatase and tensin homolog) loss, is involved in over 30 % of invasive breast cancers [63]. Signals from HER2 are transduced through the PI3K/AKT/mTOR pathway and HER2 therapies like trastuzumab inhibits signaling from these pathways to promote cell cycle arrest and apoptosis [64]. Therefore, downstream mutations or signaling dysregulation in these pathways results in acquired resistant to the treatment [16].

It has been described that several miRNAs modulate resistance to HER-therapies via PI3K/AKT/mTOR signaling pathway regulation. Ye et al. (2013) investigated the role of oncomirR - miR-221 in promoting trastuzumab resistance in HER2+ breast cancer and metastasis [65]. SKBR3 cells transfected with pre-miR221 lentiviruses showed increased cell growth when treated with trastuzumab compared to control cells. Conversely, inhibition of miR-221 in SKBR3 significantly decreased cell survival after trastuzumab treatment compared to the scrambled control. miR-221 regulates PTEN and silencing it confers trastuzumab resistance in SKBR3 cells [65]. PTEN is a tumor suppressor and loss of PTEN results in breast cancer progression and drug resistance [33]. Although a high percentage of breast cancers harbour mutations in genes that constitute the PI3K pathway and the role of the PI3K inhibitor PTEN is well documented, the incidence of PTEN gene mutations in invasive breast cancer is relatively low (less than 7 %) [66,67]. This suggests the substantial involvement of post-transcriptional gene silencing and other alterations in the PI3K pathway conferring resistance to breast cancer therapy.

In 2015, Mattos-Arruda et al. described the significant role of miR-21 overexpression in trastuzumab resistance in HER2+ breast cancer patients [68]. MiR-21 increased after trastuzumab and chemotherapy, regulating PTEN and programmed cell death 4 (PDCD4) as direct targets conferring trastuzumab and chemotherapy resistance. In addition, miR-21 signaling is involved in epithelial-to-mesenchymal transition (EMT) in HER2-overexpressing cancer cells. This can cause therapy resistance upon PI3K pathway activation via PTEN downregulation and upregulation of phosphorylated AKT (pAKT). Also, in the same year, Ma et al. (2015) discovered the contribution of miR-542-3p in modulating trastuzumab resistance in HER2+ breast cancer cell lines [69]. Of note, the study found that trastuzumab treatment can induce miRNA-5423p expression and knocking it down activated the PI3K/AKT pathway, resulting in resistance to trastuzumab. They demonstrated the role of miRNA-542-3p as a negative PI3K-AKT pathway regulator in breast cancer. Han et al. (2019) found higher levels of ERBB4 and pAKT in trastuzumab-resistant cells, which showed an increased growth rate and reduced apoptosis. Inhibition of miR-141 expression results in ERBB4 overexpression, causing trastuzumab resistance in breast cancer cells [70].

In addition, miR-375 has been implicated in the epigenetic mechanisms that mediate trastuzumab resistance by regulating insulin-like growth factor-I receptor (IGF1R) and PI3K/AKT pathways [71]. In trastuzumab-resistant cells, IGF1R interacts with HER2, forming a heterodimer, driving the drug-resistance and progression of cancer [72]. MiR-375 was predicted to target IGF1R in trastuzumab-resistant HER2+ breast cancer cells. The correlation between the downregulation of miR-375 and increased level of IGF1R was validated in clinical samples [71]. Also, miR-630 regulates cellular responses to HER2-targeted drugs, inhibiting miR630 increased drug resistance via targeting IGF1R [73]. Luo et al. (2021) demonstrated that increased IGF2 and insulin receptor substrate 1 (IRS1) activated IGF1R/AKT/mTOR signaling and promoted trastuzumab resistance in HER2+ breast cancer. This activation was regulated via FOXO3a (forkhead box O3a)- miRNA negative feedback inhibition and FOXO3a modulates miR-128-3p and miR-30a-5p to influence IRS1 protein translation [21].

### 4.2 Other mechanisms

There has been increasing evidence suggesting that cancer stem cells (CSCs), a subpopulation of cells within the tumor, can escape anti-cancer treatment [74]. Several studies were carried out to understand the mechanism of resistance modulated by miRNAs and CSCs. When investigating the role of miRNAs in regulating breast cancer stem cells (BCSC), De Cola et al. (2015) discovered the high expression of miR-205-5p that regulates ERBB2 and EGFR [75]. While ERBB2 is a direct target of miR-205-5p, EGFR regulation is mediated via p63 in BCSCs. Of note, low expression of ERBB receptors in BCSCs can lead to lapatinib resistance. Additionally, Boulbes et al. (2015) found that miR-515 plays a central role in mediating trastuzumab resistance via regulating CD44 [76]. Increasing findings are indicating the role of the TGF-β signaling pathway in breast cancer drug resistance [77,78]. Increased cancer invasiveness, metastasis and resistance to targeted therapy observed in several cancers was found to be driven by induction of EMT and TGF-β [79]. The miR-200 family was downregulated in cells undergoing EMT induced by TGF-β [80]. MiR-200c, member of miR-200 family, was significantly downregulated and inversely correlated with invasiveness and trastuzumab resistance in breast cancer cells [81]. In specific, downregulation of miR-200c can lead to TGF-β signaling activation by directly targeting zinc finger E-box binding homeobox 1 (ZEB1) and Krüppel-like zinc finger protein 217 (ZNF217). ZNF217 and ZEB1 promoted EMT via activation of TGF-β [82]; and ZEB1 transcriptionally suppressed miR-200c. Hence, the feedback signaling circuit of miR-200c/ZNF217/TGF-β/ZEB1 and miR-200c/ZEB1 resulted in miR-200c inhibition in trastuzumab-resistant cells [81].

MiR-16 can be used as a biomarker for sensitivity to HER2-targeted therapy as reduced levels are associated with resistance to radiotherapy, chemotherapy and endocrine therapy. MiR-16 inversely regulates cyclin J (CCNJ) and far upstream element-binding protein 1 (FUBP1) mediating trastuzumab and lapatinib effects [83]. CCNJ and FUBP1 are essential for the growth of HER2+ breast cancer cells and FUBP1, a transcription factor regulating transcription and translation of various genes including c-Myc [84]. C-Myc transcriptional activation can be induced by ERBB2 activation of Erk1/2 and PI3K/AKT cascades, leading to miR-16 repression and increased levels of FUBP1 and CCNJ to promote proliferation [83]. The mechanism of trastuzumab and lapatinib in HER2 targeted treatment is to block Erk1/2 and PI3K/AKT to inhibit c-Myc activation [32,48]. Hence, the upregulation of miR-16 is a biomarker for drug response in HER2+ breast cancer treated with trastuzumab and lapatinib.

Autophagy is an intracellular self-catabolic degradation process mediated by lysosomes that maintain cellular homeostasis [85]. Autophagy is considered a surviving mechanism of tumor cells under adverse treatment conditions [86]. Hence, reversing drug resistance can be achieved by targeting autophagy. Autophagy related 5 (ATG5) was the first mammalian autophagy gene identified and Han et al. (2020) reported that the knockdown of miR-567 induced trastuzumab and chemotherapy resistance by targeting ATG5 during autophagy in breast cancer [87]. Previous research has demonstrated that exosomal miR-1246 is more highly expressed in breast cancer patients compared to healthy controls [88]; and can promote cell proliferation, invasion, and therapeutic resistance by targeting cyclin G2 [89]. Zhang et al. (2020) have reported that individually, or in combination, exosomal miR-1246 and miR-155 could predict trastuzumab resistance in HER2+ breast cancer [90]. Table 1 illustrates miRNAs mediating breast cancer HER2 therapy resistance.

**Table 1:** miRNAs mediating breast cancer HER2 therapy resistance.

miRNAs	Authors	Year	Drug	Expression level	Target	Sample used	Ref.
miR-221	Ye et al.	2013	trastuzumab	up	PTEN	Cell lines/tissue	[65]
miR-21	Mattos-Arruda et al.	2015	trastuzumab	up	PTEN, PDCD4	Tissue/cell lines	[68]
miR-542-3p	Ma et al.	2015	trastuzumab	down	PI3K, AKT	Cell lines	[69]
miR-141	Han et al.	2019	trastuzumab	down	ERBB4	Cell lines/tissue	[70]
miR - 375	Ye et al.	2014	trastuzumab	down	IGF1R, AKT	Cell lines	[71]
miR-630	Corcoran et al.	2014	lapatinib, neratinib	down	IGF1R	Cell lines	[73]
miR-128-3p miR-30a-5p	Luo et al.	2021	trastuzumab	down	IRS1	Cell lines	[21]
miR-205-5p	De Cola et al.	2015	lapatinib, trastuzumab	up	P63	Tissue	[75]
miR-515	Boulbes et al.	2015	trastuzumab	down	CD44	Cell lines/tissue	[76]
miR-200c	Bai et al.	2014	trastuzumab	down	ZNF17, ZEBI	Cell lines	[81]
miR-16	Venturutti et al.	2016	trastuzumablapatinib	down	CCNJ, FUBP1	Cell lines	[83]
miR-567	Han et al.	2020	trastuzumab	down	ATG5	Cell lines/tissue	[87]
miR-1246 miR-155	Zhang et al.	2020	trastuzumab	up	Cyclin G2	Blood	[90]

## 5 miRNAs that sensitize for targeted treatment in HER2+ breast cancer

In addition to the role of miRNAs as resistance markers of HER2-therapies, studies have also uncovered their function in modulating the sensitivity of HER2-targeted therapies [[91], [92], [93]]. Jung et al. (2012) found a direct correlation between the level of plasma miR-210 and sensitivity to trastuzumab in HER2+ breast cancer patients [94]. Pu et al. (2019) reported the suppressive role of miR-135b-5p overexpression in the invasion and migration of breast cancer [95]. Later in 2020, Li et al. further discovered that miR-135b-5p could enhance the anti-proliferative and anti-metastatic effects of trastuzumab in HER2+ breast cancer. Especially, they also found that the anti-proliferative effects of trastuzumab were enhanced by miR-135–5p through downregulation of cyclin D2 [92]. In addition, miR-129-5p has also been shown to modulate trastuzumab sensitivity in HER2+ breast cancer through ribosomal protein S6 (rpS6), a downstream target of the PI3K/AKT/mTOR signaling pathway [93].

The overexpression of the EGFR family (ErbB1/HER1/HER2/HER3/HER4) induces PI3K/AKT/mTOR and signal transducer and activator of transcription 3 (STAT3) signaling pathways, contributing to drug resistance in breast cancers [96,97]. Among HER2+ breast cancer patients expressing isoform HER2δ16, about 90 % also present with metastatic disease. HER2δ16 deletion is known to affect trastuzumab binding and HER2δ16 expressing cell lines are resistant to trastuzumab [98]. MiR-7 was found to suppress both EGFR expression and the activity of HER2δ16, the oncogenic isoform of HER2, and sensitized the HER2δ16-expressing cells to trastuzumab [99].

Increased IGF1R/HER2 heterodimer formation can lead to the activation of the AKT/mTOR signaling pathway. MiR-98-5p has been found to be involved in IGF2 regulation; thus, it can modulate trastuzumab sensitivity [100]. Noyan et al. (2019) demonstrated the suppressive role of miR-770-5p in combination with trastuzumab treatment in AKT and ERK signaling. By blocking these pathways, HER2+ breast cancer cells proliferation and invasion can be inhibited [101]. Normann et al. (2022) also identified 8 miRNA mimics (miR-101-5p, mir-518a-5p, miR-19b-2-5p, miR1237-3p, miR-29a-3p, miR-29c-3p, miR-106a-5p, and miR-744-3p) sensitizing cells to targeted treatment. Among them, miR-101-5p showed the most promising effects in reducing HER2+ breast cancer cell viability and sensitizing the cells to targeted therapy including lapatinib and trastuzumab [102]. In later research, they found that miR-101-5p overexpression could inhibit HER2+ cancer cell proliferation [91].

MiR-182 has also been described as a biomarker of therapeutic response to HER2 therapy [103,104]. Sajadimajd et al. (2016) described that inhibiting miR-182 could sensitize trastuzumab-resistant HER2+ breast cancer cells via the hypoxiainducible factor 1-alpha (H1F-1α) downstream signal. Hence, trastuzumab in combination with miR-182 inhibition, can be a potential approach to suppress the HIF-1α level and boost trastuzumab sensitivity [104]. However, Yue et al. (2019) described that overexpression of miR-182 resulted in decreased trastuzumab resistance due to MET/PI3K/AKT/mTOR signaling pathway inactivation. An in vivo study demonstrated a significant tumor suppressive effect of miR-182 overexpression and trastuzumab cotreatment [103]. In addition, Tormo et al. (2017) described the role of miR-30b and miR-26a in modulating the sensitivity of HER2+ breast cancer cells to trastuzumab therapy by regulating the cyclin E2 (CCNE2) gene whose amplification/overexpression is involved in trastuzumab resistance [105,106]. A disintegrin and metalloproteinase domain-containing protein 10 (ADAM10), a primary predictor of HER2 shedding that prevents trastuzumab from binding to HER2 receptors, is a target of miR-122-5p. Increased miR-122-5p combined with trastuzumab treatment can help overcome trastuzumab resistance by suppressing ADAM 10-associated HER2 shedding [107]. Table 2 summarises the miRNAs modulating breast cancer HER2 therapy sensitivity.

**Table 2:** miRNAs modulating breast cancer HER2 therapy sensitivity.

miRNAs	Authors	Year	Drug	Expression level	Target	Sample used	Ref.
miR-210	Jung et al.	2012	trastuzumab	up	MET IGF1 MAM	Cell lines/blood	[94]
miR-135b-5p	Li et al.	2020	trastuzumab	up	cyclin D2	Cell lines	[92]
miR-129-5p	Lu et al.	2017	trastuzumab	down	rpS6	Cell lines/blood	[93]
miR-7	Huynh et al.	2014	trastuzumab	up	EGFR	Cell lines	[99]
miR-770-5p	Noyan et al.	2019	trastuzumab	up	AKT ERK	Cell lines	[101]
miR-98-5p	Zhang et al.	2021	trastuzumab	up	IGF2	Cell lines/tissue/blood	[100]
miR-101-5p	Normann et al.	2022	lapatinib trastuzumab	up	uncharacterized	Cell lines	[91]
miR-182	Sajadimajd et al.	2016	trastuzumab	down	HIF-1α	Cell lines	[104]
miR-182	Yue et al.	2019	trastuzumab	up	MET	Cell lines	[103]
miR-26a miR-30b	Tormo et al.	2017	trastuzumab	up	CCNF2	Cell lines	[105]
miR-122-5p	Sercan et al.	2015	trastuzumab	up	ADAM10	tissue	[107]

## 6 Challenges and future perspectives

Although HER2+ breast cancer accounts for a significant proportion of all breast cancer cases, there are still limited biomarkers to predict HER2-targeted therapeutic responses. This presents a considerable obstacle in identifying patients who will benefit from HER2-targeted treatment. Sample selection options and processing procedures should be carefully considered while looking for miRNA biomarkers for resistance to HER2-targeted therapies. The majority of our reviewed studies used cell lines as their study model. This can bring some benefits regarding the availability of samples for multiple experiments and the reduced cost. However, validation with clinical samples should be confirmed to reflect the nature of miRNAs in HER2+ breast cancer drug resistance patients. Another challenge in this field is the lack of reproducibility across studies. As observed in the list of our reviewed studies, a minimal overlap is seen in the identified miRNA panels among studies. This represents the complicated biology of miRNA expression in tissues and the circulatory system of HER2+ breast cancer patients. Moreover, a majority of the published articles focused on studying miRNAs in trastuzumab resistance, and limited or no studies have been conducted for other HER2-targeted drugs, such as neratinib or tucatinib.

In the future, we expect a higher number of studies focusing on miRNA biomarkers in HER2-targeted treatment and their significance in identifying suitable treatments for HER2+ breast cancer patients. Further standardization and improvement in sampling techniques might help introduce more reliable results. Additionally, rapid development of bioinformatics technologies, machine learning and advanced tools will facilitate data analysis and functional downstream analysis. Many trastuzumab-resistance studies have discovered the dominant roles of specific miRNAs in distinctive models; however, to thoroughly understand the resistance mechanisms, crosstalk between signaling events regulated by these miRNAs should also be considered in future studies. Finally, more effort will be made to understand the mechanism of miRNAs in resistance and reverse-resistance of HER2 treatment to choose an appropriate therapeutic method for HER2+ patients or enhance the sensitivity of existing HER2-targeted drugs.

## 7 Conclusion

The identification of miRNA biomarkers regulating targeted drug resistance in HER2+ breast cancer plays a vital role in directing HER2+ breast cancer patients to appropriate treatment options. In addition, by understanding the functions and mechanisms of miRNAs that sensitize cancer cells to HER2 therapy, potential therapeutic strategies can also be developed for poor HER2 therapy respondents. The main contribution of this study is the establishment of a thorough taxonomy of functions and mechanisms of miRNAs in the study of HER2+ breast cancer drug resistance and modulation. Given substantial breakthroughs in miRNA biomarker research in breast cancer over the past years, the number of studies on HER2-treatment resistance is still limited. We, therefore, believe that research in miRNAs regulating HER2 treatment resistance still holds many aspects to develop in the future.

## Ethics approval and consent to participate

Not applicable.

## Consent for publication

Not applicable.

## Availability of data and materials

Not applicable.

## Funding

This work is supported by funds from South East Technological University, President’s Scholarship (WD-2022-14-WSCH).

## CRediT authorship contribution statement

**Thanh Hoa Vo:** Conceptualization, Methodology, Formal analysis, Data curation, Writing – original draft, Writing – review & editing, Visualization. **Esam EL-Sherbieny Abdelaal:** Validation. **Emmet Jordan:** Validation. **Orla O'Donovan:** Validation. **Edel A. McNeela:** Formal analysis, Writing – review & editing, Validation. **Jai Prakash Mehta:** Validation. **Sweta Rani:** Conceptualization, Methodology, Investigation, Data curation, Writing – original draft, Writing – review & editing, Supervision, Funding acquisition.

## Declaration of competing interest

The authors declare that they have no known competing financial interests or personal relationships that could have appeared to influence the work reported in this paper.

## Data Availability

Data will be made available on request.
